# Adaptive immunity and vaccination – iron in the spotlight

**DOI:** 10.1093/immadv/ltab007

**Published:** 2021-06-17

**Authors:** Alexandra E Preston, Hal Drakesmith, Joe N Frost

**Affiliations:** 1 MRC Human Immunology Unit, MRC Weatherall Institute of Molecular Medicine, University of Oxford, John Radcliffe Hospital, Oxford, UK; 2 Haematology Theme, Oxford Biomedical Research Centre, Oxford, UK

**Keywords:** iron, hepcidin, adaptive immunity, vaccination

## Abstract

Vaccination programmes are critically important to suppress the burden of infectious diseases, saving countless lives globally, as emphasised by the current COVID-19 pandemic. Effective adaptive immune responses are complex processes subject to multiple influences. Recent genetic, pre-clinical, and clinical studies have converged to show that availability of iron is a key factor regulating the development of T and B cell responses to infection and immunisation. Lymphocytes obtain iron from circulating transferrin. The amount of iron bound to transferrin is dependent on dietary iron availability and is decreased during inflammation via upregulation of the iron-regulatory hormone, hepcidin. As iron deficiency and chronic inflammatory states are both globally prevalent health problems, the potential impact of low iron availability on immune responses is significant. We describe the evidence supporting the importance of iron in immunity, highlight important unknowns, and discuss how therapeutic interventions to modulate iron availability might be implementable in the context of vaccination and infectious disease.

## Introduction – iron deficiency, inflammation, and hepcidin

Iron is an essential cofactor in cellular biochemistry and its catalytic function is thought to be intrinsically linked to the evolution of life [1, 2]. In the form of Fe-S clusters, haem groups, and individual ions, the element supports diverse processes, including energy production via the electron transport chain, DNA replication and repair, oxygen sensing, and demethylation reactions [3–5]. However, both iron deficiency and iron overload are prevalent across the global human population, illuminating iron as a particularly interesting micronutrient in human biology.

The importance of considering iron in the context of immunity lies with the vast prevalence of iron deficiency, particularly in countries heavily burdened by infectious disease. As recorded in 2016, it is estimated that 1.2 billion people worldwide have iron deficiency anaemia [6]. It is a leading cause of years lived with disability in low–middle-income countries (LMICs), and the fourth leading cause globally, particularly affecting children and pre-menopausal women. Our appreciation of the burden of iron deficiency is limited by an overwhelming focus on recording the prevalence of anaemia. Iron deficiency in the absence of anaemia (resolved by low ferritin) is estimated to be even more prevalent, and indeed, iron deficiency impacts on human biology beyond erythropoiesis (e.g. growth and cognitive development [7, 8]). Recent work indicates particular scenarios and populations in whom many cell types may experience a high degree of iron limitation, based on measuring serum iron concentration and transferrin saturation. In high-income settings, serum iron levels in healthy individuals vary relatively little by age, and are generally 10–30 µmol/L [9]. In The Gambia, the prevalence of anaemia among infants is high, and these children also frequently present with extremely low serum iron levels (below 5 µmol/L for much of the first year of life) [10]. Given the well-established high prevalence of iron deficiency anaemia among infants from LMICs, these observations of low serum iron in Gambian infants are likely generalisable to infants in other LMIC settings.

Central to understanding the pathophysiology of iron deficiency is an appreciation of homeostatic iron control by the hepatic iron regulatory hormone, hepcidin. Through its ability to target ferroportin (the iron exporter) for degradation [11], hepcidin maintains iron homeostasis by dictating the location of iron. In conditions of excess iron, hepcidin expression is induced and ferroportin is degraded on enterocytes and erythrophagocytic red-pulp macrophages, preventing dietary iron absorption and the recycling of iron derived from phagocytosis of senescent erythrocytes, respectively. Conversely, inhibition of hepcidin (e.g. during erythropoietic demand or iron deficiency [12, 13]) promotes iron absorption and releases iron from macrophage and hepatocyte storage, thus augmenting serum iron concentration. Yet hepcidin is not only a hormone which homeostatically regulates iron, but also an acute phase response protein. Its expression is induced by inflammation, predominantly via the cytokine, IL-6 [14]. Inflammatory induction of hepcidin is essential for the hypoferremia response to acute inflammation in mouse models [15, 16] and has been observed to correlate with inflammatory hypoferremia in a number of human infections [17–20]. Such hepcidin-mediated hypoferremia has been suggested as a form of nutritional immunity, essential for the control of extracellular siderophilic bacterial pathogens [21, 22].

Systemic iron deficiency can be nutritional in origin, driven by iron-poor diet or diets enriched with factors that antagonise iron absorption [23]. However, systemic iron availability can also be ‘functionally’ low due to chronically raised hepcidin in the context of inflammation, restricting duodenal iron absorption and iron availability in the serum, regardless of sufficiency of ferritin iron stores [24]. Functional iron deficiency can arise secondary to infection, and is common in areas where infection rates are high [25–27]. The global burden of iron deficiency is driven by the combination of poor nutritional uptake (or absorption) and high hepcidin driven by inflammation.

Iron deficiency is not limited to LMICs. In high-income countries, infants, children, pregnant women, and individuals with chronic inflammation are at particular risk of iron deficiency [8, 28]. Worldwide, 20% women of reproductive age and ~40% pregnant women are anaemic [29], with the latter often leading to neonatal iron deficiency [30]. Reportedly, menstruation affects iron stores to a greater extent than dietary intake [31]. In the pathophysiological contexts of chronic inflammation [e.g. cancer, chronic kidney disease (CKD), obesity or autoimmunity], raised hepcidin limits systemic iron availability, contributing to widespread anaemia of inflammation [32–38]. Serum iron levels can also be particularly low in conditions of gastrointestinal disease. For example, coeliac disease poses both systemic inflammation and disrupted gut epithelial integrity, impeding iron absorption [39, 40].

Thus, iron deficiency is commonly nutritional or secondary to infection and inflammation, with a proportion of cases manifesting in anaemia. Many population studies measure rates of iron deficiency anaemia (often defined by low haemoglobin and ferritin concentrations), which likely do not accurately identify individuals with serum iron deficiency. As we will describe, the latter parameter is the more relevant measure from the perspective of iron influencing adaptive immunity.

## Serum iron and adaptive immune responses

Adaptive immune responses require dynamic cellular reconfiguration of metabolism and cellular physiology as antigen-specific lymphocytes proliferate, acquire effector functions, and generate immunological memory [41]. Activated T cells express 1 million new copies of transferrin receptor (TFRC) within 24 hours of activation [42], suggesting that increased iron uptake is required to power the T cell response, consistent with the multiple roles of iron for cellular metabolism [1, 2].

Strong genetic evidence for a role of iron in human adaptive immunity comes from analysis of members of two families with severe immunodeficiency and susceptibility to infection, who were shown to carry a hypomorphic mutation in TFRC, encoding transferrin receptor 1 [43]. This mutation reduces the efficiency with which immune cells can uptake transferrin-bound iron from serum. These patients had normal numbers of T, B, and NK cells, but lacked circulating IgG and had reduced numbers of circulating memory B cells. Furthermore, *ex vivo* T and B cell proliferation was defective, but could be rescued by provisioning supraphysiological amounts of elemental iron, thus bypassing the defect in transferrin receptor. Mice with an analogous mutation in TFRC exhibit similar *ex vivo* and *in vivo* lymphocyte activation defects [42]. These results are consistent with previous studies in animal models and *in vitro*, which indicate the importance of transferrin-bound iron uptake for lymphocyte activation [44, 45]. While complete inhibition of transferrin-bound iron uptake blocks lymphocyte development [46, 47], the effect of more subtle changes in iron availability on lymphopoiesis remains unclear [42, 43, 48].

The study by Jabara *et al*.[43] demonstrates that adaptive immune responses require iron, however, the influence of variable serum iron availability on immune cells remained unaddressed. Our group recently demonstrated that transient acute serum iron deficiency, driven physiologically through enhanced hepcidin activity, suppresses the antigen-specific CD8, CD4, and B cell immune response in mice [42]. Furthermore, in piglets, a natural model of iron deficiency, we found that responses to vaccination were improved by iron interventions that increased serum iron [42]. These results are consistent with earlier observations of impaired humoral immunity and baseline lymphocyte activation *ex vivo* in rodent models of severe dietary iron deficiency [48, 49]. They also highlight serum iron, regulated by hepcidin, as the factor controlling immune responses. Consistent with this concept, we observed decreased vaccine-inducible pathogen-specific antibody responses in human patients with rare mutations that cause high hepcidin and low serum iron [42].

## Inflammatory hypoferremia – benefits and risks

Hepcidin-mediated hypoferremia is a natural part of the acute infection response [50–52]. Hepcidin is commonly elevated during infection and inflammation, preventing macrophagic iron recycling and ‘trapping’ the micronutrient in reticuloendothelial cells, limiting the systemic availability of iron and its potential to support pathogen growth. However, the aforementioned data regarding the iron requirements of lymphocytes imply the existence of a trade-off between hypoferremia as an innate nutritional defence to a subset of infections [50, 53] and the metabolic requirements of the adaptive immune response [42]. The outcome of this trade-off likely depends on the pathogen in question, the persistence of the hypoferraemic response and the quantity and distribution of iron resources within the host. For example, while iron deficiency certainly protects against malaria [54] and certain bacterial infections [21, 22, 50, 55, 56], there is no clear evidence that iron availability directly alters viral replication *in vivo* [57].

**Figure 1.**
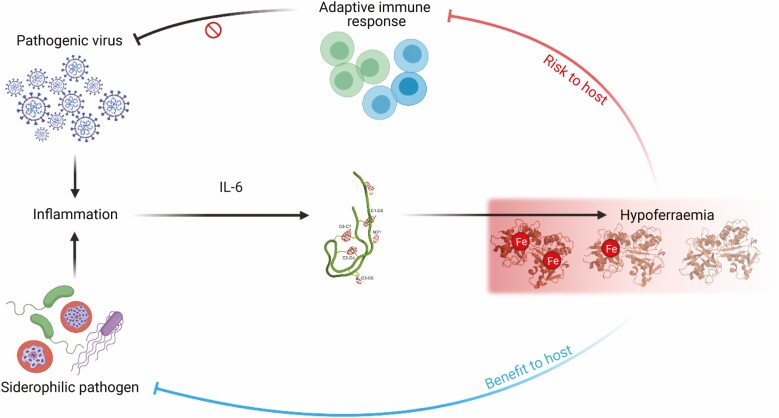
Inflammation, often resulting from infection, induces hepcidin expression via IL-6, driving hypoferraemia. Hypoferraemia poses a trade-off to the host, limiting extracellular iron availability and protecting against siderophilic pathogens (e.g. *Vibrio vulnificus*), but also diverting iron away from the adaptive immune response, impairing control of pathogenic viruses (e.g. Influenza A virus infection).

Viruses can induce hypoferraemia in humans, as noted in experimental norovirus infection and acute HIV-1, however, in these instances, the effect may be transient [17, 19]. The COVID-19 pandemic has provided a context in which to investigate how hypoferremia and adaptive immunity may be associated in humans in a more chronic systemic inflammatory setting. Inflammatory hypoferremia has been associated with severe disease and low lymphocyte counts among patients with COVID-19 [58–63]. Emerging evidence indicates that uncoordinated or slowed development of effective adaptive immunity may be a driver of severe disease [64–66]. Interestingly, given the role of IL-6 in systemic iron homeostasis during inflammation [14], its expression is elevated in cases of acute COVID-19 and associates with disease severity [63, 67, 68]. IL-6 levels have also been suggested to correlate inversely with various aspects of the effector T cell response [65]. Clinical trial results indicate that therapeutic blockade of IL-6 with drugs such as tocilizumab and sarilumab may improve patient outcome [69, 70]. Whether disrupted iron homeostasis associates with impaired adaptive immunity in COVID-19, and how this might be approached therapeutically, remains to be investigated. However, inducing inflammatory hypoferremia in a murine model of influenza infection impaired adaptive immunity, delayed viral clearance, worsened the severity of lung inflammation, and slowed recovery from weight loss [42]. This indicates that hypoferremia may predispose to poor outcomes of respiratory infection. This outcome exists in stark contrast to the protective effects of hepcidin-driven hypoferraemia in the context of extracellular bacteria and liver-stage malaria [21, 22, 53]. A potential explanation for this divergence is that denying iron to siderophilic pathogens benefits the host. However, if the iron requirements of the pathogen are relatively low (such as for viral replication), inflammatory hypoferraemia instead risks inhibiting a protective adaptive immune response, allowing infection to persist and potentially exacerbating tissue damage due to unrestrained inflammation (Fig. 1). Notably, this scenario suggests that suppressing hepcidin activity could provide a therapeutic target for restoring iron availability to activated lymphocytes in inflammatory settings.

## Iron deficiency and vaccine responses in humans

When considering studies seeking to link iron status and responses to vaccines in humans, as previously reviewed by Oppenheimer [49], early investigations tended to provide inconsistent results. Vaccination trials for diseases such as diphtheria, tetanus [71], and typhoid [72] did not indicate an association between iron deficiency and vaccine efficacy, but were likely underpowered [73]. A larger study involving 1554 Ecuadorian infants showed that anaemic children generate low diphtheria antibody titres (frequently below the protective level) following DTP vaccination compared with control [74]. More recently, anaemia was linked to altered development of the immune system in children from Mozambique and Tanzania [75]. It was recently demonstrated that variable combinations of anaemia, haemoglobin, and soluble transferrin receptor measurements in Kenyan infants predicted poor responses to diphtheria, pertussis, measles, and pneumococcal vaccines [76]. However, anaemia is a multifactorial condition, which can be driven by influences other than iron deficiency [77]. Direct evidence that serum iron deficiency regulates responses to vaccines in humans is limited because this parameter is rarely reported. One small study found that among elderly hospitalised patients receiving influenza vaccines, non-response was clearly associated with suppressed serum iron concentrations (mean, 8.34 µmol/L vs. 16.00 µmol/L in responders) [78]. More significantly, iron supplementation at time of vaccination, which would be expected to raise serum iron, improved the antibody response to the measles vaccine in Kenyan infants [76].

Lower vaccine efficacy has been observed for certain vaccines, including measles and live attenuated influenza vaccine, among infants in LMICs [79, 80]. Certain high-income populations, including individuals with coeliac disease [81], obesity [82], and CKD [83] are reported to generate poor responses to the hepatitis B virus vaccine, and the inactivated flu vaccine has been reported to perform poorly in CKD patients [84] and the elderly [85]. However, whether the high prevalence of iron deficiency and low serum iron in these populations contributes to low vaccine efficacy has not yet been explored, to our knowledge.

## Iron and immunometabolism

Iron deficiency has been shown to have broad effects on metabolism and function of different types of cells [86–91]. In general, cellular iron homeostasis is maintained by iron-regulatory protein-1 and -2 [92]. Loss of the genes encoding these proteins in activated T cells impairs iron uptake, proliferation, and effector functions [42]. However, the precise iron-requiring processes disrupted in T cells by iron-restricted conditions, critically impairing activity, remain unclear. *In vitro*, both low-iron culture conditions and genetic disruption of tetrahydrobiopterin synthesis (which impacts T cell proliferation in an iron-dependent manner) impair T-cell mitochondrial oxidative metabolism [42, 93]. DNA and histone demethylases are iron-dependent, and modulation of their activity through iron chelation results in altered cell cycle behaviour in B cells [48, 94]. Serum iron deficiency prevents acquisition of effector functions by T cells [42]. In CD4 T cells, iron controls expression of proinflammatory cytokines IL-2 and GM-CSF (but not IFNγ and TNFα) via interactions with the RNA binding protein, PCBP1 [44]. Iron has also been suggested to modulate the responsiveness of lymphocytes to IL-2R signalling [42, 95]. The effects of low iron on T cells are likely to be complex and there may be different levels of sensitivity depending upon the iron requirements and metabolic states of different T cell subsets.

Transient serum iron deficiency during the expansion phase of the T cell response impairs the quality of CD8 T cell memory 35 days post-immunisation (assessed by cytokine production and magnitude of secondary recall response) [42]. This result suggests that iron availability may influence the trajectory of T cell differentiation and polarisation of the immune response. However, more work is required to establish how the negative effects of iron deficiency persist over time, and how the cell-intrinsic and extrinsic regulators of memory differentiation are thus perturbed [96].

Although this review focuses on iron deficiency, we should note that genetic diseases which suppress hepcidin (hereditary hemochromatosis and thalassemia [97]) and lead to systemic iron loading have also been proposed to influence immunity. In HFE hemochromatosis patients, this impact is complicated by the strong association between HFE mutations and the major histocompatibility complex gene complex. The effect of HFE mutations on baseline CD8:CD4 ratios and lymphocyte activation phenotypes has been extensively investigated [98–100], yet it is not well understood how HFE mutations alter the quality of adaptive immunity *in vivo*.

## Correcting iron deficiency to improve immunity – challenges and potential

**Figure 2.**
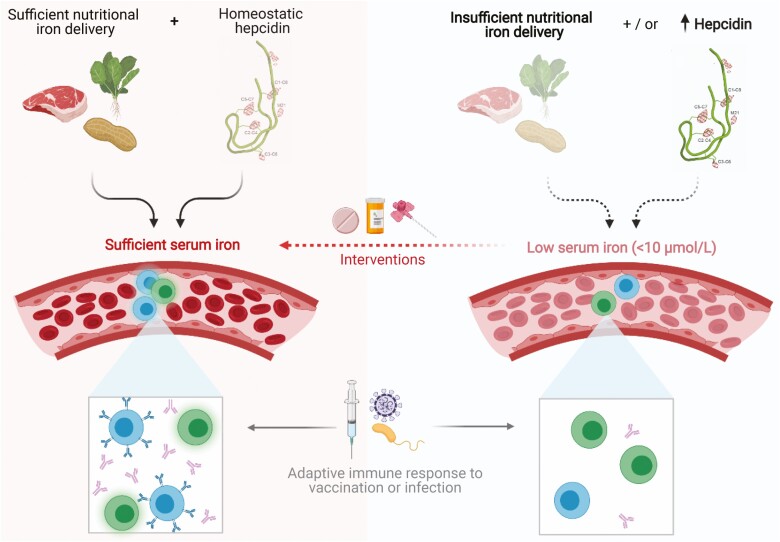
Dietary factors and constitutively high hepcidin expression can drive low serum iron availability. Sufficient iron concentrations are required to generate a robust adaptive immune response. In particular settings, it may be possible to acutely supplement serum iron availability to support immunity, thereby improving vaccine efficacy – this concept remains to be investigated.

The suggestion that iron deficiency may contribute to suboptimal adaptive immunity makes the normalisation of iron status an attractive potential ‘immunotherapeutic’ intervention (Fig. 2). However, the key evidence that therapeutically targeting iron can support adaptive immunity is currently limited to the improved T cell and antibody responses seen in hypoferraemic mice and piglets, respectively, upon iron supplementation [42], and the improved seroconversion and antibody avidity to measles vaccination in a retrospective analysis of Kenyan infants receiving iron [76]. It is critically important that prospective randomised controlled trials are performed to more rigorously test the idea that iron could boost immune responses to vaccines in iron-deficient individuals, and research in this area is ongoing.

Of relevance to this concept, the resolution of iron deficiency remains a challenge in itself for several reasons. Oral iron formulations may exacerbate some infections, including malaria, and can increase episodes of diarrhoea, although these results are not universally observed in all settings [101–106]. Furthermore, the efficacy of oral iron in restoring iron status can be variable. A major issue in populations with high infection burden is that persistent mild inflammation can drive high hepcidin concentrations, disabling efficient dietary iron absorption [107, 108]. High-dose oral iron supplementation regimes may also increase hepcidin to a point where the majority of iron is not absorbed [109, 110]. Future improvements in dosing schedules [111–113], new iron formulations that are more absorbable [114, 115], and potentially the use of intravenous iron [116], could counteract the current difficulties associated with nutritional iron interventions. Other molecular mechanistic approaches, aimed at inhibiting hepcidin and so enhancing iron absorption and release of iron from cellular sequestration [117–121], may also be beneficial, particularly in the context of inflammatory anaemias.

Despite the unresolved issues noted above, one specific consideration for the possible future use of iron to improve immune responses is worth highlighting: less iron is likely to be needed to boost immune responses than is needed to improve systemic iron deficiency. The latter often requires iron interventions over long periods, with oral iron regimes lasting for several months. The nature of this exposure (time and quantity) likely contributes to the noted gastrointestinal side effects and predisposition to infections. However, to achieve the goal of enhancing the immune response to specific vaccines, only short-term supplementation during the metabolically active acute expansion phase of adaptive immunity may be necessary, as quiescent memory cells are likely to have low iron requirements. As each red blood cell contains ~100-fold more iron atoms than a T cell [122–124], it is likely that less iron administration would be required to support an immune response than for longer-term resolution of anaemia. Furthermore, over two thirds of the total body iron content exists in the erythroid compartment [123], highlighting that the number of red blood cells required to sufficiently increase haemoglobin during anaemia greatly exceeds the number of antigen-specific T cells generated at the peak of even a large immune response [125]. Acute iron treatment likely presents less infection risk than long-term oral-iron regimes, although accompanying short-term prophylactic measures may be required to further reduce infection risk.

## Concluding remarks

Driven by our increasing knowledge of systemic iron homeostasis and immunity, iron has been revealed as an important immunometabolite. Serum iron status influences the adaptive immune response to infections and vaccinations. The protective innate immune effect of low serum iron in the context of extracellular siderophilic bacterial infections is offset by the possible impairment of adaptive immunity. Our knowledge of hepcidin regulation could potentially be leveraged to swing this risk–benefit balance in favour of improving immunity. The highly variable nature of serum iron status warrants deeper research into which immune cell types have low iron levels in the varying contexts of systemic iron deficiency outlined above. The high global prevalence of iron deficiency, particularly in populations at risk of poor vaccine responses, highlights the hypothetical value of therapeutically optimising serum iron levels to improve vaccination efficacy. Trials will be required to ascertain which vaccines are particularly impaired by iron deficiency and could be improved by iron supplementation, the optimal iron delivery regime for maximising a durable vaccine response, and in which populations we should target iron interventions.

## Data Availability

No new data were generated or analysed in support of this research.

## Abbreviations

CKD: Chronic kidney disease
LMIC: Low- and middle-income countries
TFRC: Transferrin receptor
